# Diphtheria and Nephritis

**Published:** 1883-08

**Authors:** 


### DIPHTHERIA AND NEPHRITIS.

WE are still in want of observations which shall throw light on the nature of the renal changes associated with diphtheria. Our readers have been kept *au courant* with the latest writings on the subject, and are therefore fully acquainted with the doctrine of the infective nature of the nephritic alteration. It is philosophical to bear in mind that albuminuria, so often attendant on cases of diphtheria, may not necessarily be due to actual disease of the kidneys; the manifold antecedents or causes of albuminuria may still come into play, even although the body be the subject of diphtheria. The presence of one cause, however probable its action may be, does not preclude the possibility of a less striking factor being the real agent in any particular instance. We are led to make the foregoing remarks, because Dr. Furbringer, in Virchow's *Archiv* for March, has been unable to detect the micrococci which other observers have demonstrated in the urine and kidneys of cases of diphtheria. We are far from asserting a full belief in the view that diphtheritic nephritis is even generally a bacterial disease; on the contrary, the notion that the primary diphtheritic disease is due to micrococci requires more convincing evidence than has at present been adduced. Dr. Furbringer has investigated the clinical history and morbid anatomy of diphtheritic nephritis with much industry. He comes to the conclusion that the renal changes may conveniently be arranged under three divisions, according to the degree of anatomical change. The earlier stages are characterized by comparatively slight changes in the epithelium of the cortical region, not unlike "cloudy swelling"; next the cellular degeneration becomes more decided and more widely spread; and there are some alterations in the interstitial tissues, but no vascular lesions. The final degree is comparable with that of the intensely congested kidneys sometimes seen in connection with nephritis after scarlet fever.

---

ACCORDING to the *Milling World*, sackcloth or canvas can be be made perfectly impervious to moisture, equal to leather, by steeping it in decoction of one pound of oak bark with fourteen pounds of boiling water. The cloth has to soak twenty-four hours, when it is taken out, passed through running water, and hung up to dry. This quantity is sufficient for eight yards of stuff. The flax and hemp fibers, in absorbing the tannin, are at the same time better fitted to resist wear. This recipe is useful to millers who sack flour.

